# Automated Hematoma Detection and Outcome Prediction in Patients With Traumatic Brain Injury

**DOI:** 10.1111/cns.70119

**Published:** 2024-11-12

**Authors:** Yang Xu, Qiuyu Fu, Mengqi Qu, Junyao Chen, Jianqi Fan, Shike Hou, Lu Lu

**Affiliations:** ^1^ School of Disaster and Emergency Medicine Tianjin University Tianjin China; ^2^ Georgia Tech Shenzhen Institute Tianjin University Tianjin China; ^3^ College of Intelligence and Computing Tianjin University Tianjin China

**Keywords:** classification, deep learning, mortality prediction, segmentation, traumatic brain injury

## Abstract

**Purpose:**

To develop a tool for automated subtype classification and segmentation of intracranial hemorrhages (ICH) on CT scans of patients with traumatic brain injury (TBI). Furthermore, outcome prediction for patients can effectively facilitate patient management.

**Methods:**

This study presents a cascade framework for two‐stage segmentation and multi‐label classification. The hematoma region of interest (ROI) is localized, and then the ROI is cropped and resized to the original pixel size before being input into the model again to obtain the segmentation results. In multilabel classification, the mask obtained from automatic segmentation is superimposed onto the corresponding ROI and CT slices, respectively, to constitute the input image. Subsequently, the ROI image is employed as the local network input to obtain local features. Third, the CT image is utilized to construct a feature extraction network to obtain global features. Ultimately, the local and global features are fused dimensions in the pooling layer, and calculated to generate the final retrieval results. For the prediction of 14‐day in‐hospital mortality, automatically extracted hematoma subtype and volume features were integrated to enhance the widely used CRASH model.

**Results:**

The proposed segmentation method achieves the best estimates on the Dice similarity coefficient and Jaccard Similarity Index. The proposed multilabel classification method achieved an average accuracy of 95.91%. For mortality prediction, the best model achieved an average area under the receiver operating characteristic curve (AUC) of 0.91 by 5‐fold cross‐validation.

**Conclusions:**

The proposed method enhances the precision of hematoma segmentation and subtype classification. In clinical settings, the method can streamline the evaluation of ICH for radiologists, and the automatically extracted features are anticipated to facilitate prognosis assessment.

## Graphical abstract

Our study aims to automatically classify and segment CT images of patients with traumatic brain injury using a deep learning model. Based on the automatic classification and segmentation, volume and subtype characteristics of the hematoma were extracted and combined with other clinical information to predict in‐hospital mortality.

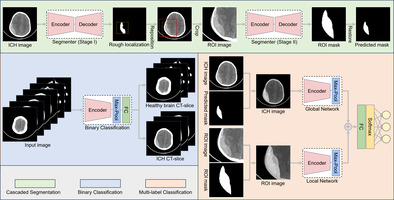

## 1 Introduction

Traumatic Brain Injury (TBI) refers to a severe injury to the brain produced by a forceful external force, leading to potential temporary or permanent malfunction of the brain's neurological functions. It has become a widespread and significant neurological condition on a global scale [1, 2, 3], affecting approximately 70 million individuals [4, 5]. Patients who cannot receive prompt treatment following an intracranial hemorrhage (ICH) may experience long‐term disability or possibly mortality. Therefore, timely and accurate diagnosis and management of ICH in patients with TBI is essential to reduce poor patient outcomes.

Computed tomography (CT) is the main imaging method used to initially screen for ICH since it is widely accessible and provides quick results [6, 7, 8]. Utilizing multimodal data to diagnose TBI is a focus of research to make it more relevant for therapeutic purposes [9, 10]. In addition, determining the subtype and volume of ICH using medical imaging plays a crucial role in prognostic prediction and in diagnosing if surgery is required for TBI patients [11, 12, 13, 14, 15]. Practically, the widespread use of CT imaging may require clinicians to commit a significant amount of time and effort. Physicians of different qualifications may produce different results when detecting and manually delineating ICH [16]. Therefore, implementing automated classification and segmentation of CT images will help resolve these disparities.

Determining the specific type of ICH is a crucial part of the treatment process. ICH can be divided into five categories: intraparenchymal hemorrhage (IPH), intraventricular hemorrhage (IVH), epidural hemorrhage (EDH), subdural hemorrhage (SDH), and subarachnoid hemorrhage (SAH). Moreover, CT images of most patients with TBI are usually diagnosed as containing one or more ICH subtypes. Initial studies on the automated classification of CT slices from TBI were limited to identifying hemorrhages [17, 18, 19, 20]. With the introduction of precision medicine and increased clinical needs, a more practical approach is to correlate medical images with multiple lesion labels [21]. Several recent research have suggested techniques for differentiating between multiple types of ICH, which can aid in the automated identification of hemorrhage [16, 22, 23]. While these methods can help to automatically detect hemorrhage, they do not go further to calculate hematoma volume and thus do not provide accurate quantitative results. To address this issue, the researchers proposed methods to detect subtypes while segmenting hematomas [24, 25, 26, 27, 28]. The Dice coefficient of these approaches was approximately 0.59, indicating that their performance still needed to be further improved.

CT images have challenges in the classification process due to their significant inter‐class similarity, making it difficult to differentiate between lesion regions and tiny lesions. A single deep global feature alone is insufficient for retrieval to accurately represent the information contained in medical images, which compromises the method's robustness and classification performance. Moreover, depending solely on simple categorization labels may not sufficiently direct the network's attention toward important areas, thus constraining model performance. During the segmentation process, extracting global characteristics of the CT image as a representation may contain a significant amount of noise, as the ICH region only occupies a small percentage of the image. Therefore, there is still a need for further enhancement in the segmentation performance. In terms of prediction, the contribution of automated detection and estimation of CT scans to outcome prediction has not been fully explored.

In this study, an improved deep learning method was proposed to enhance the detection performance of ICH. To further explore the contribution of hematoma type and volume to outcome prediction, this study trains a machine learning algorithm for mortality classification by combining clinical information and features extracted from automated hematoma detection. The principal contributions of this work are as follows:

1. A cascaded segmentation and multi‐label classification framework is proposed to reduce the amount of computation by filtering the input of nonbleeding CT slices, thereby improving the efficiency of the model.
2. To enhance the precision of CT image segmentation for small lesions, a straightforward and efficient tandem two‐stage segmentation approach is presented, which helps to extract more image features.
3. A strategy of reusing self‐predicted segmentation maps is used to increase the utilization of lesion masks, thereby improving the performance of classification tasks.
4. To solve the problem of missing hematoma, features are extracted from the global network and local network as a complete information representation, thus improving the accuracy of retrieval results.

## 2 Materials and Methods

### 2.1 Datasets

In this study, there are three datasets of cerebral hemorrhage used to train and evaluate the proposed method. The two publicly available datasets are RSNA Intracranial Hemorrhage Detection [29] and PhysioNet [30]. The private dataset collected by our team is named MS‐TBI and has received ethical approval from the Ethics Committee of the Fourth Central Hospital of Tianjin, China (Ethics Code: SZXLL‐2023‐KY028). A detailed description of the datasets can be found in the Supporting material.

### 2.2 Image Preprocessing and Augmentation

In the pretraining of the classifier models, we trained them on the RSNA 2019 Brain CT Bleeding Challenge dataset and tested it using the PhysioNet dataset. In the training and validation of the segmenter model, we used the PhysioNet and MS‐TBI datasets.

**FIGURE 1:**
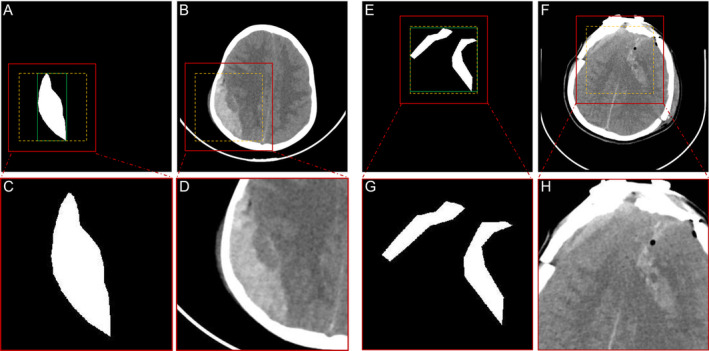
Examples of annotated images. (A) and (E): GT of input segmentation Stage I; (B) and (F): CT slices of input segmentation Stage I; (C) and (G): Mask of input segmentation Stage II; (D) and (H): ROI squares of input segmentation Stage II.

The CT slices were uniformly resized to a pixel size of 512×512 when creating the training and validation sets for the segmentation and classification tasks. The example images used to train the cascaded two‐stage segmentation model (named Stage I and Stage II) are shown in Figure 1. Ground truth (GT; Figure 1A,E) and CT slices (Figure 1B,F) are used for training the segmentation Stage I. Mask (Figure 1C,G) and CT slices (Figure 1D,H) were cropped from the region of interest (ROI) obtained using GT to train the segmentation Stage II.

The detailed steps for data production for input Stage II are as follows: (1) Obtain the CT slice and the corresponding ground truth. (2) Locate the smallest square within the boundaries of all ROIs according to the GT (green box region) and adjust to square boxes (yellow box region). (3) Extend the square box outward by 20 pixels to get the image inside the ROI (red box area). (4) Crop this area and resize it to the pixel size of the original image.

The reason we chose to externalize the ROI region by 20 pixels is that in most cases, the grayscale value of the hemorrhage is different from the grayscale value of the surrounding normal brain tissue. Therefore, the background information (the tissue surrounding the hemorrhage) has a positive effect on segmentation.

To enhance the data, horizontal flip, vertical flip, rotation (magnitude of ±45°), zoom (magnitude of 80%–120%), pan (magnitude of ±20%), and shear (magnitude of 0%–10%) were taken with a randomization rate of 50%.

### 2.3 Proposed Method

**FIGURE 2:**
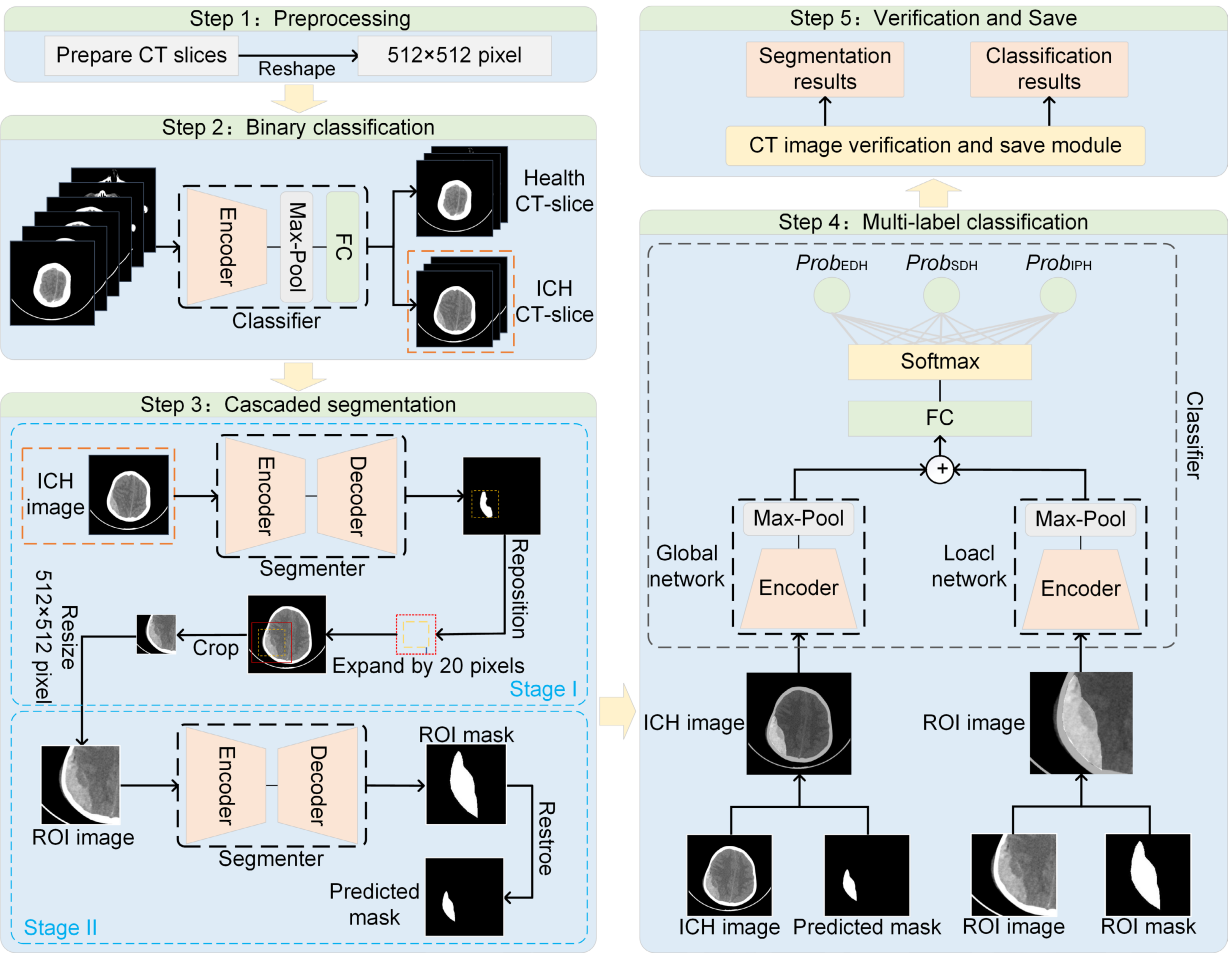
Our proposed network architecture consists of three parts.

The objective of this study is to assess the type and volume of hemorrhage. Then, the contribution of these features to outcome prediction is explored. Herein, the framework proposed in this paper consists of a binary classifier, a multi‐label classifier, and a two‐stage segmenter in series. The flowchart of our method is shown in Figure 2. The details of each step are presented in the next section.

Convolutional Neural Networks are used to realize the task of segmentation of medical images [31]. The representative network models are DeepLab [32] and UNet [33]. Among them, DeepLabV3+ can obtain richer contextual information through multiple scales to improve the accuracy of segmentation. UNet is a network with an encoder‐decoder structure, which is designed to better solve the medical image segmentation problem. Therefore, these two network models are selected in this paper to compare and prove the effectiveness of the proposed tandem two‐stage segmentation method.

The classical ResNet [34] and the latest EfficientNet [35] have been chosen for the backbone network for comparison. The reasons for the selection include the ability of ResNet to extract complex and detail‐rich medical image features and its superior generalization ability on a variety of image datasets. EfficientNet achieves higher accuracy on multiple benchmark datasets, and its design principle allows the model to capture more key features in medical images, thus improving the classification accuracy.

#### 2.3.1 Multi‐class Segmenter

Two cascade networks with the same encoder–decoder structure are trained using the dice loss function. First, CT slices with hemorrhages are detected using a binary classifier, which is fed into the segmentation model. The first segmentation network (Stage I) is trained to provide a coarse localization of the hemorrhage region (also known as the region of interest, ROI), and based on the coarse localization, the second segmentation network (Stage II) is trained to perform fine segmentation. Pre‐experiments show that the contextual information provided in Stage I may not play a significant role in the refinement of Stage II. Therefore, we only use the images within the ROI obtained by ground truth to train Stage II. Finally, a voting mechanism is used to obtain the final prediction map. The voting mechanism is detailed in Figure S1.

#### 2.3.2 Multi‐label Classifier

For the multilabel classification task on CT scans, we propose a classification model that integrates global and local features. First, the CT image is superimposed with the mask provided by the segmenter and subsequently fed into the global network for processing, thereby enabling the extraction of global features. The ROI image, overlaid with the mask generated by the segmenter, is employed as the input for the local network to extract local features. Finally, the global and local features are integrated dimensionally and undergo classification to achieve the multi‐label classification task.

### 2.4 Mortality Prediction

Inclusion criteria included patients with moderate‐to‐severe TBI (msTBI), a Glasgow Coma Scale (GCS) score of 3–12, and age in the range of 18–80 years. Patients were excluded if they had previous neurosurgical disease or cancer, did not have an admission CT head scan before neurosurgical intervention, or if they lacked outcome information. One hundred and fifty‐one patients were included in the study, which included clinical information, the first admission CT scan, and masks created and verified by a radiologist. Table 1 lists the demographic and baseline clinical characteristics of these patients.

**TABLE 1:** Characteristics of patients with msTBI.

Characteristics	MS‐TBI dataset(*n* = 151)
Median age in years (IQR)	57.5 (49.8–66)
No. of males (%)	117 (77.5)
Cause of injury, no. (%)	
Traffic accidents	53 (35.1)
Falls or drops	40 (26.5)
Assault	7 (4.6)
Others or unknown	51 (33.8)
Median GCS score (IQR)	6.5 (3–10)
Median risk of 14‐day coma (%)	17 (11.3)
Median risk of 14‐day mortality (%)	46 (30.5)
Pupils react to light, no. (%)	
Both nonreactive	50 (33.1)
One reactive	25 (16.6)
Both reactive	76 (50.3)
Major extra‐cranial injury, no. (%)	18 (11.9)
Petechiae, no. (%)	123 (81.5)
Effaced basal cistern, no. (%)	14 (9.3)
Midline shift, no. (%)	28 (18.5)
Evacuated hematoma, no. (%)	95 (62.9)
Type of bleeding, no. (%)	
EDH	47 (31.1)
IPH	8 (5.3)
IVH	14 (9.3)
SAH	136 (90.1)
SDH	142 (94.0)
Median length of hospitalization, days (IQR)	21 (7–37)

Abbreviations: EDH, epidural hematoma; GCS, glasgow coma scale; IPH, intraparenchymal hematoma; IQR, interquartile range; IVH, intraventricular hemorrhage; SAH, subarachnoid hemorrhage; SDH, subdural hematoma.

Many clinical observations are associated with mortality in patients with TBI. The CRASH model, which assesses in‐hospital 14‐day mortality in patients, has been widely used and validated [36, 37, 38, 39, 40]. There are two types of CRASH models: the CRASH‐BASIC (Corticoid Randomization After Significant Head Injury) and the CRASH‐CT (CRASH‐basic extended by computed tomography features). The CRASH‐BASIC model included features such as age, GCS, Pupils' reaction to light, and Major extra‐cranial injury. The CRASH‐CT adds diagnostic information from CT images to the base model, including the presence of petechial hemorrhages, obliteration of the third ventricle or basal cisterns, subarachnoid bleeding, midline shift, and nonevacuated hematoma. In this study, we aimed to investigate the predictive validity of adding hematoma volume and hemorrhagic subtype characteristics to the CRASH model. Specifically, we aimed to assess whether the proposed model is a good predictor of in‐hospital mortality at 14 days.

In this study, we reconstructed the above models using logistic regression and random forest methods, respectively. We evaluated the predictive ability of the proposed model against the baseline model.

## 3 Results

### 3.1 Experimental Setup

All models were implemented in Python (version 3.8.15) programming language and Pytorch (version 1.12.1) deep learning framework. The hardware and software configurations of the system include CPU: Intel(R) Core (TM) i7‐11,700 @ 2.50 GHz, RAM: 32.0 GB, GPU: NVIDIA GeForce RTX 3080, OS: Windows 10 Professional (version 22H2).

### 3.2 Performance Comparison of the Binary Classification

Evaluating classification performance using accuracy. We employ the ResNet and EfficientNet architectures as the backbone networks for feature extraction. ResNet‐18, ResNet‐34, ResNet‐50, and EfficientNet B2 to B6 models, which were initialized with pretrained weights, underwent training for 100 epochs using the RSNA dataset. The test results indicate that six sets of network models may attain a maximum accuracy of 99%. Accordingly, the most efficient baseline models, ResNet‐50 and EfficientNet B6, were selected for the segmentation and muti‐label classification tasks.

### 3.3 Performance Comparison of Segmentation

In measuring the performance of segmentation, we use the Intersection over Union (IoU) and Dice coefficient.

The initial learning rate of the model is 1e‐4, the number of iterations is 500, the learning rate adjustment strategy is CosineAnnealingLR, the optimizer is Adam, and the loss function is Dice Loss. ResNet‐50 and EfficientNet B6 described in Section 3.2 were chosen for the backbone network, and the segmentation architecture is selected as DeepLabV3+ and UNet. The comparison results are all averaged for five‐fold cross‐validation. Table 2 shows the segmentation performance of the proposed segmentation method compared to other baseline methods. It can be seen that segmentation Stage II shows a significant performance improvement compared to segmentation Stage I. In addition, the proposed segmentation method outperforms the ICH‐UNet [30] baseline method in both Dice and IoU metrics in these four common network combinations, with a maximum improvement of about 49% and 47% compared to ICH‐UNet.

**TABLE 2:** Comparison of segmentation assessment metrics between two‐stage segmentation models and baseline methods.

Network	Stage I or II	PhysioNet dataset		MS‐TBI dataset	
		Dice	IoU	Dice	IoU
ICH‐UNet	—	0.3150	0.2180	0.6332	0.5765
DeepLabV3+ (Encoder: EfficientNet‐B6)	Stage I	0.7461	0.6280	0.9038	0.8571
	Stage II	**0.7999**	**0.6823**	**0.9279**	**0.8812**
DeepLabV3 + (Encoder: ResNet‐50)	Stage I	0.6798	0.5656	0.8761	0.8097
	Stage II	**0.7969**	**0.6788**	**0.9266**	**0.8798**
UNet (Encoder: EfficientNet‐B6)	Stage I	0.7607	0.6448	0.9027	0.8585
	Stage II	**0.8031**	**0.6856**	**0.9268**	**0.8806**
UNet (Encoder: ResNet‐50)	Stage I	0.7128	0.5965	0.8808	0.8294
	Stage II	**0.7925**	**0.6730**	**0.9261**	**0.8743**

*Note:* The bold data denotes the best value.

**FIGURE 3:**
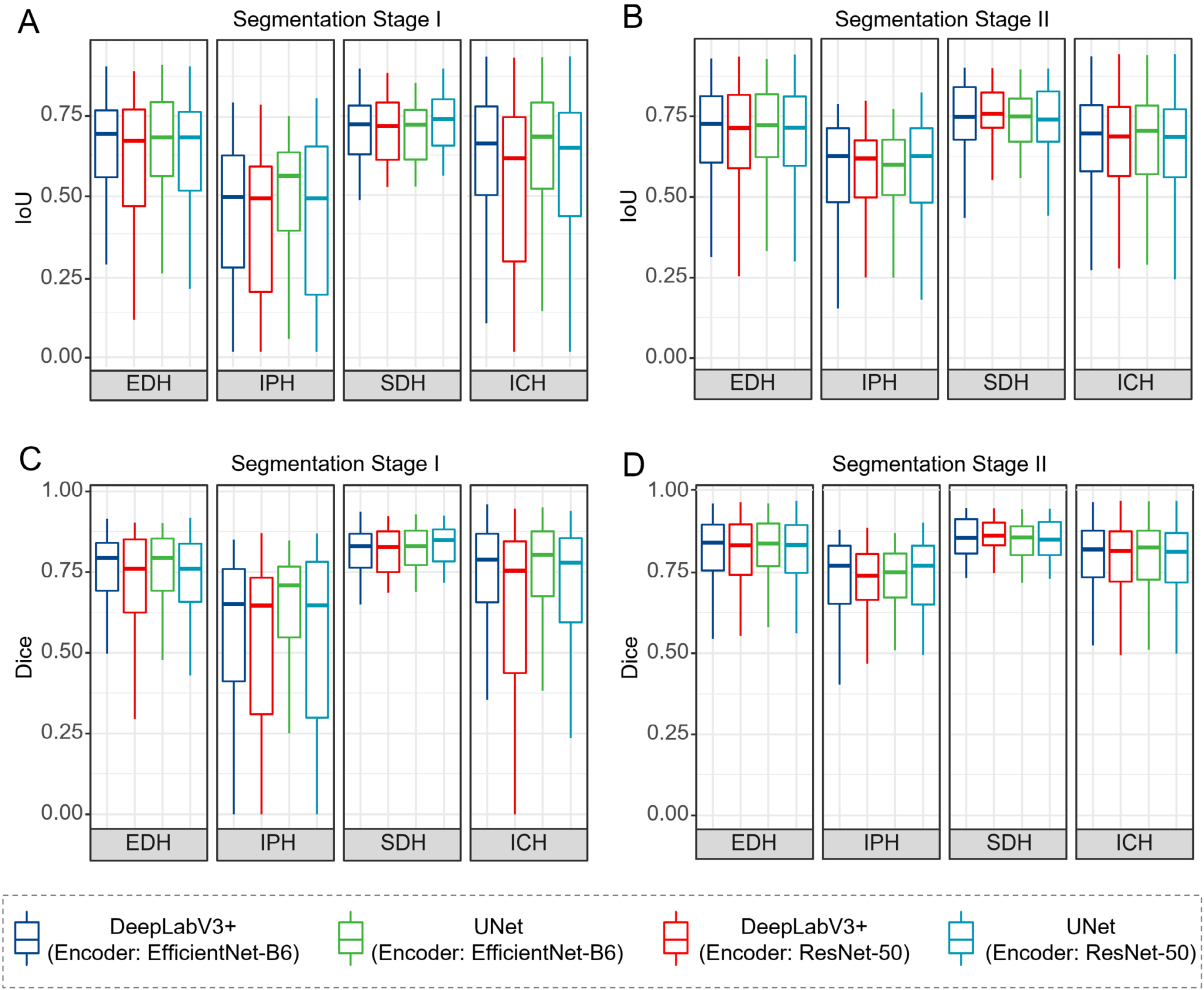
Boxplot of IoU and Dice metrics for ICH and its subtypes under the PhysioNet dataset.

Figure 3 shows the comparison of Dice and IoU metrics between segmentation Stage I and Stage II. Only three subtypes, EDH, SDH, and IPH, which are of particular importance for emergency surgical decisions [24, 41], are presented in the figure. It can be seen that all bleeding subtypes performed better in segmentation Stage II than in Stage I, and the spacing between the upper and lower quartiles of the different ICH subtypes was shorter, indicating more focused results. In conclusion, these comparisons demonstrate that the performance of segmentation can be improved by using a two‐stage cascade architecture.

**FIGURE 4:**
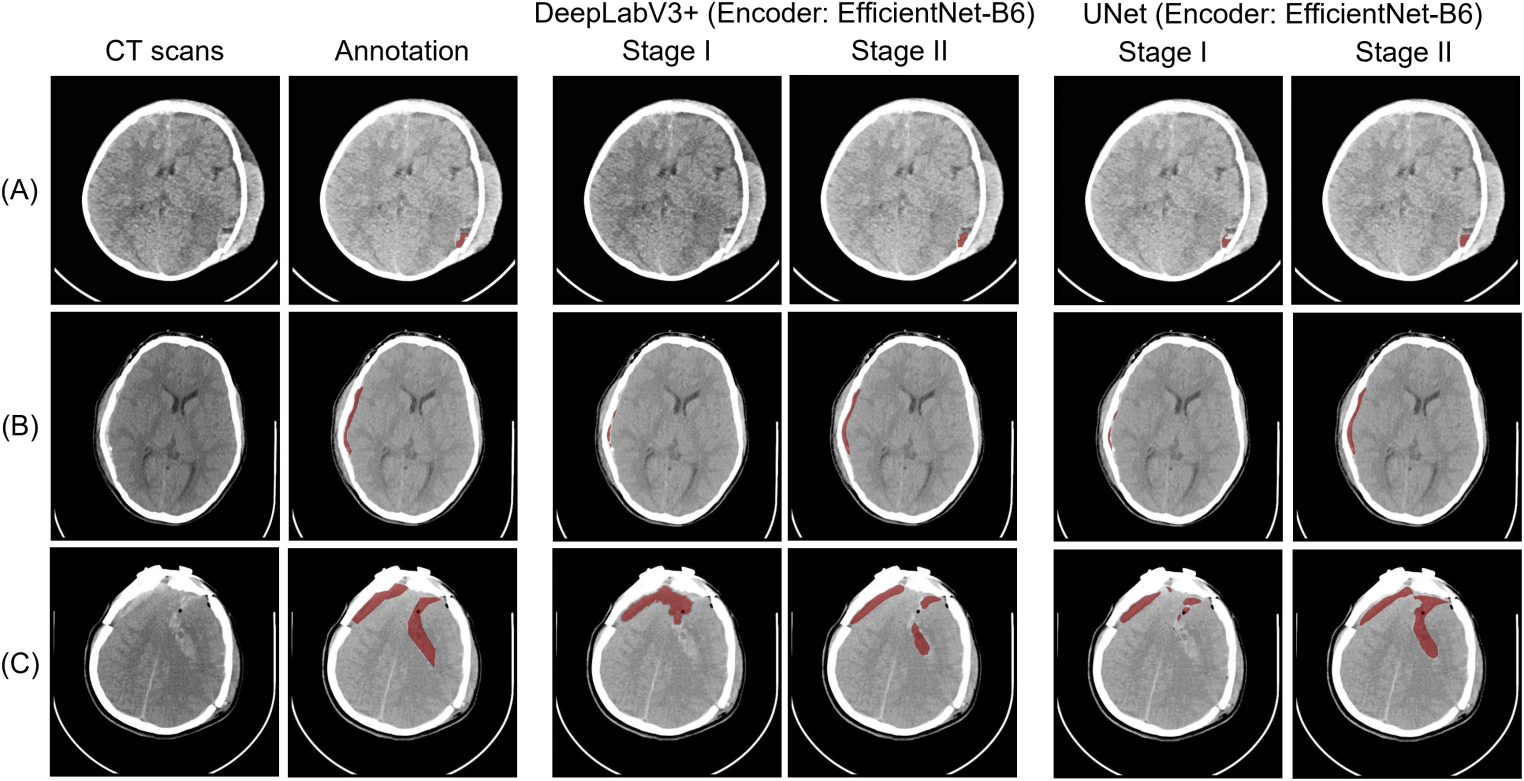
Comparison of two‐stage segmentation methods on the PhysioNet dataset. Annotated and predicted hematomas are shown in red.

**FIGURE 5:**
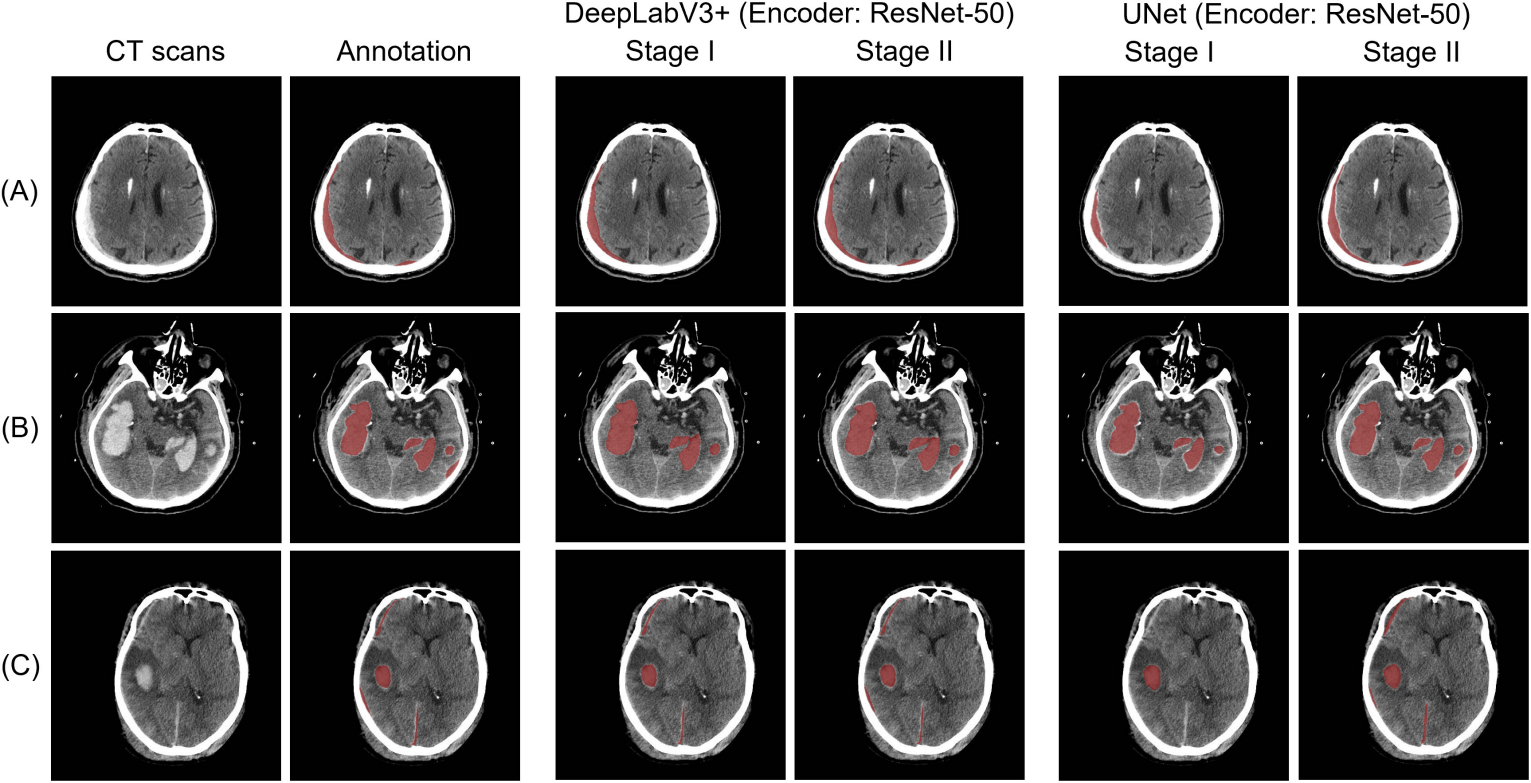
Comparison of two‐stage segmentation methods on the MS‐TBI dataset. Annotated and predicted hematomas are shown in red.

Figure 4 shows the prediction results of our method (encoder: EfficientNet B6) on the PhysioNet dataset. Figure 5 shows the prediction results on the MS‐TBI dataset using the ResNet‐50 backbone network. The results show that the proposed method obtains ideal segmentation results with very high sensitivity regardless of the amount of hemorrhage.

### 3.4 Performance Comparison of Multi‐label Classification

To evaluate the results of the categorization output, we used the following five metrics, including Accuracy, Precision, Precision, Recall, F1‐score, and AUC.

**FIGURE 6:**
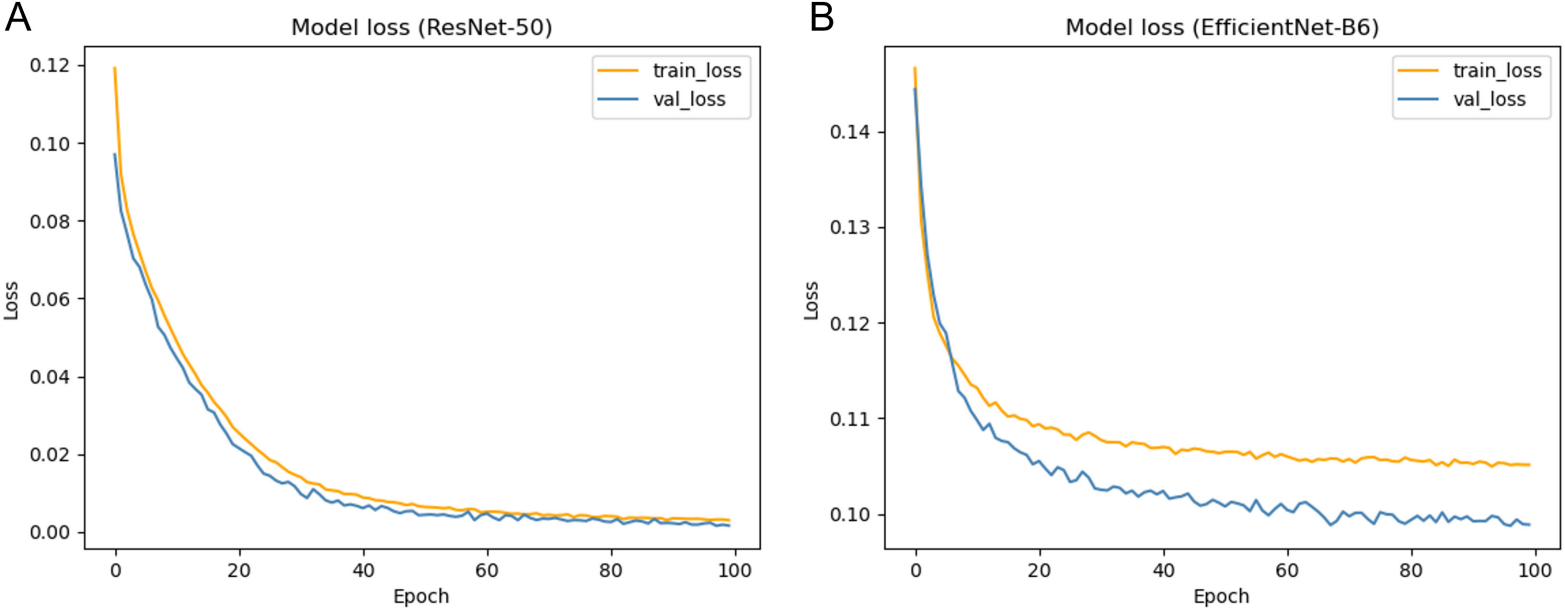
(A): Loss curves for training and validation under the ResNet‐50 model; (B): Loss curves for training and validation under the EfficientNet‐B6 model.

The learning rate is 1e‐4, the iteration setting is 100, and the loss function used is focal Loss. The comparison results are all averaged for five‐fold cross‐validation. Figure 6 illustrates the continuous decrease of training and validation loss with the increasing number of iterations under the ResNet‐50 and EfficientNet‐B6 models. Table 3 compares the ICH‐UNet baseline method, which has surpassed the baseline methodology in all metrics except for the lower Recall score. It is noteworthy that the ResNet‐50 network yielded the most favorable outcomes, with an accuracy of 95.91%. In addition, different indicators were compared for five types of hemorrhage (Table S1).

**TABLE 3:** Performance comparison results of the proposed multi‐label classification method on the PhysioNet dataset.

Network	Accuracy	micro‐Precision	micro‐Recall	micro‐F1	micro‐AUC
ICH‐UNet	0.5656	0.2310	**0.9710**	0.3732	0.5347
EfficientNet‐B6	0.9536	0.7859	0.6644	0.7082	0.9373
ResNet‐50	**0.9591**	**0.8370**	0.7094	**0.7679**	**0.9596**

*Note:* The bold data denotes the best value.

### 3.5 Performance Comparison of Mortality Prediction

For mortality prediction, we used the AUC, F1‐score, sensitivity, specificity, and precision for model evaluation. A five‐fold cross‐validation strategy was used for all models to avoid bias introduced by the data.

We designed two schemes A and B for the outcome of patients. A: Death is categorized as one category, and other conditions are categorized as one category. B: Death and coma are categorized as one category, and other conditions are categorized as one category.

We employed the Box‐Tidwell test to evaluate the assumption of linearity between continuous predictors and the log odds in the logistic regression model. The results indicated a nonlinear relationship between age and the log‐odds of the outcome (*p* = 0.0350), while other continuous variables, including GCS (*p* = 0.5746) and hemorrhage volume (*p* = 0.2911), were consistent with the linearity assumption. To improve the model's fit and generalizability, a Box‐Cox [42] transformation was applied to the age variable.

**FIGURE 7:**
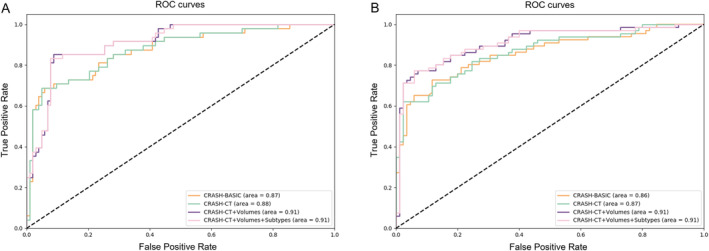
Graphs of AUC under two scenarios under the logistic regression model. (A): AUC for the scheme A; (B): AUC for the scheme B.

Table 4 shows the 14‐day in‐hospital mortality prediction results for each model. It can be seen that in both scenarios based on logistic regression and random forest algorithms, the model (CRASH‐CT + Volumes + Subtypes) that includes both hematoma volume features and subtype features outperformed the baseline model. Both scenarios A and B showed that both hematoma volume and hematoma subtypes were important predictors of mortality. Figure 7 illustrates the AUC plots based on the logistic regression algorithm for both scenarios, showing that the models had the highest AUC values when both hemorrhage volume and subtype information were included.

**TABLE 4:** Comparison of 14‐day mortality predictions across models.

Model	Logistic regression				Random forest			
	Accuracy	Precision	Recall	F1	Accuracy	Precision	Recall	F1
A. Mortality								
CRASH‐BASIC	0.7815	0.6207	0.8178	0.7025	0.7524	0.7385	0.6867	0.6966
CRASH‐CT	0.7682	0.6029	0.7956	0.6841	0.8095	0.7497	0.7489	0.7409
CRASH‐CT + Volumes	0.8011	0.6383	**0.8578**	0.7274	0.8000	0.7432	0.7667	0.7498
CRASH‐CT + Volumes + Subtypes	**0.8075**	**0.6478**	**0.8578**	**0.7341**	**0.8190**	**0.7751**	**0.7689**	**0.7688**
B. Mortality (including death and coma)								
CRASH‐BASIC	0.7742	0.7292	0.7857	0.7548	0.7905	0.8575	0.7429	0.7929
CRASH‐CT	0.7746	0.7292	0.8022	0.7619	0.8190	0.8417	0.7890	0.8125
CRASH‐CT + Volumes	0.8206	0.7832	0.8165	0.7993	0.8762	**0.9203**	0.8341	0.8731
CRASH‐CT + Volumes + Subtypes	**0.8340**	**0.7918**	**0.8473**	**0.8178**	**0.9048**	0.9092	**0.8802**	**0.8922**

*Note:* The bold data denotes the best value.

**FIGURE 8:**
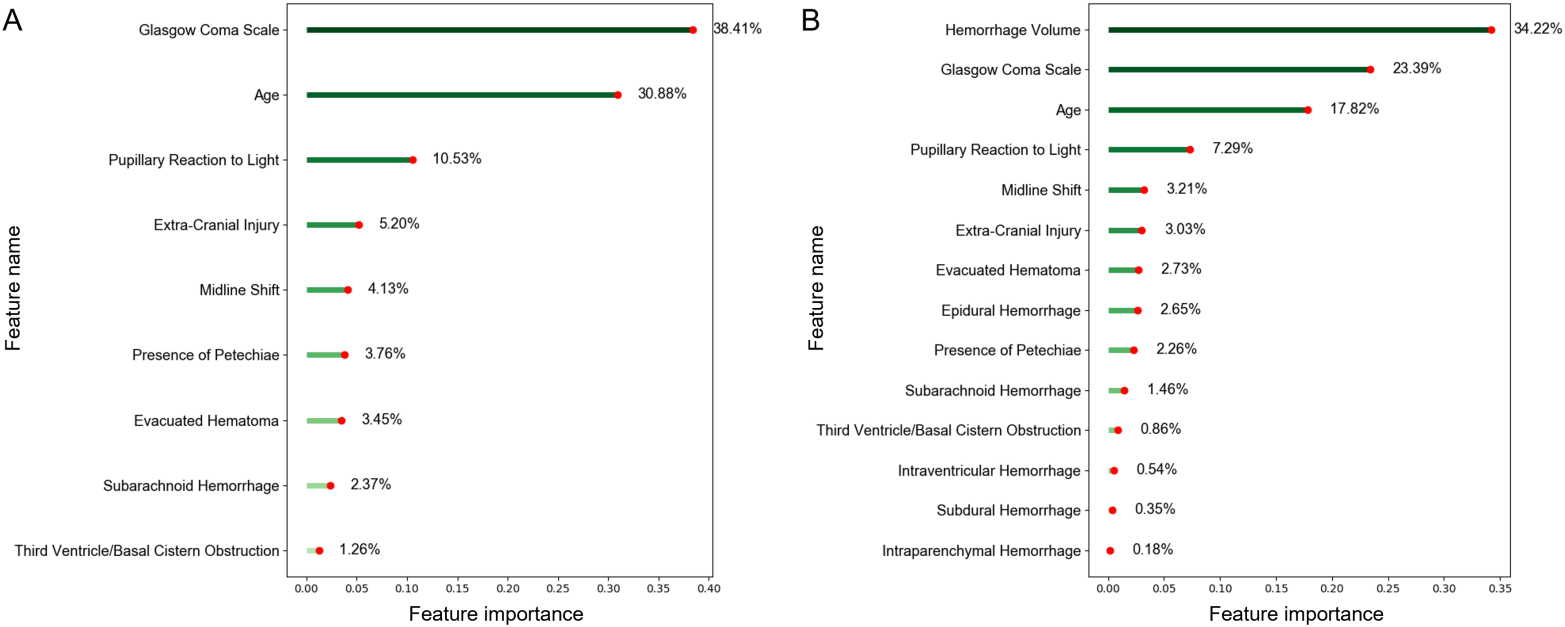
Ranking of feature importance based on the random forest model. (A): Feature importance of “CRASH‐CT”; (B): Feature importance of “CRASH‐CT + Volumes + Subtypes”.

We computed feature priority rankings using the random forest model. Figure 8 illustrates the relative significance of features in the “CRASH‐CT” model compared to the “CRASH‐CT + Volumes + Subtypes” model. The ranking of “CRASH‐CT” reveals that CT performances such as SAH, Petechiae, and Midline shift hold significant value. Nevertheless, in the “CRASH‐CT + Volumes + Subtypes” ranking, hematoma volume obtained the highest rank, representing 34.22% of the importance, significantly surpassing the significance of the following features. The ranking of feature relevance suggests that systems utilizing automated quantitative measurements can offer insights into the association between hematoma and death prediction, making them a useful addition.

## 4 Discussion

Neurotrauma physicians often make timely and personalized decisions about whether patients with TBI require surgery upon admission. The task of CT image annotation can be time‐consuming and labor‐intensive, and to assist in this process, we have built a deep learning‐based assisted diagnosis process (Figure 2).

The advantage of transferring only CT slices with hemorrhages to the next task is that it not only reduces the computational load on the model but also allows the model to avoid unhelpful parameter tuning due to pseudo‐false positives during training.

Considering the characteristics of CT images, pathologically unimportant regions will account for the vast majority of the region, and clinically significant information is highly concentrated in small regions of the image. The advantage of this is that by cropping out the ROI region, the training can be focused on the key region, reducing the amount of computation in image processing and improving the processing efficiency. At the same time, ROI can also help the model extract the target content from the complex background for subsequent analysis and processing. In other fields, such as the segmentation of pericardial adipose tissue [43], kidney tumors [44], and carotid artery vessel wall [45], the segmentation method using cropped ROI as a priori guiding knowledge proved to be effective. Therefore, we adopted the same strategy for hematoma segmentation in TBI. As seen from the results in Table 2 and the segmentation comparisons (Figures 4 and 5), in different network structures and datasets, performance is improved by a minimum of 2.4% and a maximum of 11.7% compared to no prior guidance knowledge. Our proposed method of two‐stage segmentation exhibits superior performance, further highlighting the effectiveness of emphasizing ROI areas. This integration enables more precise depiction of small hemorrhage regions, which is critical for improving clinical diagnosis and decision‐making.

By reviewing the literature, Anna Guan et al. [46] proposed an accurate medical image hash retrieval method combining interpretability and feature fusion for chest X‐ray images. Erdal Ozbay et al. [47] used the same method to improve the classification accuracy of brain tumors. This proves that the feature fusion approach has significant advantages in medical image retrieval. Based on this, we utilize the segmentation map predicted by the segmentation module as the guiding knowledge for classification and then fuse the global and local features with the superimposed mask to form our proposed multilabel classification model. The results show that this improvement can be attributed to our model's ability to better capture both local and global features, which is crucial for accurately delineating hemorrhagic regions, especially when the lesions are small or diffuse (Table 3).

The clinical outcome of TBI is closely related to its hemorrhage volume and location, and different subtypes can also reflect different locations. It has been experimentally demonstrated that computer‐assisted assessment of ICH is more predictive of functional outcome and mortality than the standard ABC/2 method [48]. In this study, we compared our model to the widely used CRASH model in 151 patients with msTBI (Table 4). The results demonstrated that our model outperformed the CRASH model, which can be attributed to the inclusion of automatically extracted hematoma subtype and volume features—variables not considered in the CRASH model. These features, combined with clinical indicators, may serve as potential markers for the early determination of patient mortality.

While there is currently limited direct quantitative evidence demonstrating the impact of automated image analysis tools on patient outcomes, prior studies have shown that reducing diagnostic time and minimizing manual intervention can lead to faster treatment initiation, which is a key factor in improving patient prognosis in acute conditions such as traumatic brain injury and stroke [49, 50]. Our tool could reduce radiologist workload and speed up the decision‐making process by automatically extracting hematoma subtypes and volumes, which will contribute to these clinical improvements.

## 5 Limitations

The work also has limitations in its interpretation of the results. While our proposed method enhances classification and segmentation accuracy, there is still a need for additional improvement in segmentation performance, particularly in handling small hemorrhages and distinguishing between closely related tissue types. In addition, image annotations can also affect the performance of the model. We will also continue to explore ways to improve the quality of the data to further improve the reliability and accuracy of the model in future studies.

Given that the mortality prediction results were solely validated on a single dataset, it is imperative to get additional external datasets for further corroborative verification in subsequent studies. Subsequent endeavors should persist in gathering fresh patient data from multiple institutions to ensure the robustness and generalizability of the model. Additionally, exploring more advanced deep learning architectures and integrating multimodal data, such as prior knowledge in the field of imaging, could further improve segmentation and prediction performance.

## 6 Conclusion

In this study, we propose a cascade framework of multi‐label classification and two‐stage segmentation for quantitative assessment of CT in patients with TBI. Our method exhibits superior performance to methods proposed in previous studies, thus demonstrating great potential for clinical development. In a clinical setting, the application of this fully automated method could reduce the time spent by radiologists for image segmentation and evaluation of hematomas, thereby potentially benefiting patient care by accelerating the surgical decision‐making process. In addition, the volumetric and subtype features extracted from the automated tool characterize the hematoma better than the qualitative and semi‐quantitative features assessed manually, and the established predictive model is expected to assist physicians in predicting the survival outcomes of patients with TBI.

## Conflicts of Interest

The authors declare no conflicts of interest.

## Supporting information


Data S1.

## Data Availability

The datasets analyzed during the current study are available from the corresponding author upon reasonable request. The code for the algorithm in question is publicly available on GitHub: https://github.com/LuckyXuYang/Prediction‐of‐mortality‐in‐TBI‐patients.
