# Supplementary material for: Automated Hematoma Detection and Outcome Prediction in Patients With Traumatic Brain Injury
Source: CNS Neurosci Ther. 2024 Nov 12;30(11):e70119. doi: 10.1111/cns.70119 (PMC11557439; doi:10.1111/cns.70119)
Supplement: Supplementary file 1 — Data S1. [file CNS-30-e70119-s001.docx]

**Supplemental Information**

**Datasets**

- **RSNA 2019 brain hemorrhage challenge dataset**

The Radiological Society of North America (RSNA) dataset can be found in the Kaggle Challenge and is used to identify ICH subtypes. The dataset with annotations was collected and compiled by three research institutions located in the north and south of America. More than 25000 CT scans, totaling 755948 slices in PNG format providing a pixel size of $512\times512$, each of which may have multiple ICH subtypes.

- **PhysioNet**

The PhysioNet dataset repository collects head CT scan images of 82 subjects with TBI, including 46 male and 36 female scans, with an average age of 27.8 years. Each CT scan contained approximately 34 slices with a slice pixel size of $650\times650$. Two radiologists recorded the type of hemorrhage and produced ground truth for 2814 CT slices.

- **MS-TBI**

This research has been granted ethical approval from the Ethics Committee of the Fourth Central Hospital of Tianjin, China (Ethical Code: SZXLL-2023-KY028). The MSTBI dataset incorporates head CT and clinical data from 151 patients with moderate to severe TBI. Each patient had a single CT of approximately 56 slices with a slice thickness of 2.5 mm. A total of 4967 CT slices with hemorrhages in PNG format of $512\times512$ pixels were collected and diagnosed by radiologists for hemorrhage type and mask labeling.

**Voting mechanism**

The voting mechanism represents an intuitive and effective approach to integrated learning. The fundamental objective is to enhance the overall performance by integrating the prediction outcomes of multiple models. For each iteration of cross-validation, the convolutional neural network model converges to a distinct value based on the optimized weights. Therefore, to obtain the best results, the optimized weights obtained from the training and validation processes in each fold are used, instead of selecting a single ideal weight among them. Figure S1 depicts the representation of the voting mechanism under five-fold cross-validation. Each voxel generates a weight matrix for the result of each fold, wherein the values (0 and 1) represent the acceptance or rejection of the result. The voting process culminates in a final decision, which is based on a super-majority vote (3/5) for the voxel.


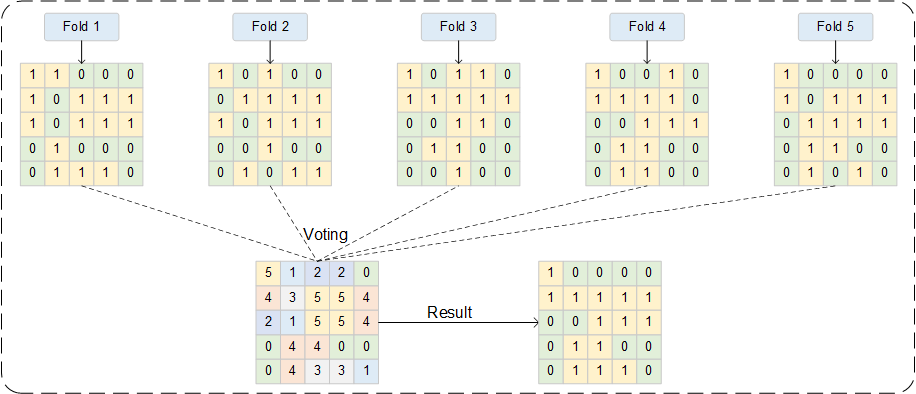


Figure S1. Voting mechanism employed to make decisions about voxels, whether hemorrhage and non-hemorrhage, by generating a weight matrix for each of the five folds using two different colors, namely, green and yellow with values 0 and 1, respectively that corresponds to the rejection or acceptance of the voxel.

Table S1. Comparison of metrics of the proposed multi-label classification method for different subtypes on the PhysioNet dataset.

| **Method** | **Type** | **Accuracy** | **macro-Precision** | **macro-Recall** | **macro-F1** | **macro-AUC** |
| --- | --- | --- | --- | --- | --- | --- |
| EfficientNet-B6 | EDH | 0.9362 | 0.7930 | 0.5809 | 0.6570 | 0.7892 |
|  | IPH | 0.9759 | 0.7959 | 0.7485 | 0.7699 | 0.9274 |
|  | IVH | **0.9936** | **0.8589** | 0.7698 | **0.8079** | **0.9841** |
|  | SAH | 0.9655 | 0.5755 | 0.7069 | 0.5896 | 0.9010 |
|  | SDH | 0.8970 | 0.6049 | **0.8445** | 0.6520 | 0.9245 |
|  | Average | 0.9536 | 0.7256 | 0.7301 | 0.6953 | 0.9052 |
| ResNet-50 | EDH | 0.9395 | **0.8796** | 0.5772 | 0.6970 | 0.8129 |
|  | IPH | 0.9727 | 0.7607 | 0.7468 | 0.7537 | 0.9409 |
|  | IVH | **0.9928** | 0.8543 | 0.7075 | **0.7740** | **0.9556** |
|  | SAH | 0.9840 | 0.5755 | 0.6334 | 0.6031 | 0.8863 |
|  | SDH | 0.9840 | 0.5834 | **0.8580** | 0.6945 | 0.9415 |
|  | Average | 0.9591 | 0.7307 | 0.7046 | 0.7045 | 0.9074 |

Note: The bold data denotes the best value.
